# Assessing Topographical Orientation Skills in Cannabis Users

**DOI:** 10.1100/2012/137071

**Published:** 2012-01-03

**Authors:** Liana Palermo, Filippo Bianchini, Giuseppe Iaria, Antonio Tanzilli, Cecilia Guariglia

**Affiliations:** ^1^Dipartimento di Psicologia, “Sapienza” Università di Roma, 00185 Rome, Italy; ^2^Sezione di Neuropsicologia, IRCCS Fondazione Santa Lucia, 00179 Rome, Italy; ^3^Departments of Psychology and Clinical Neurosciences and Hotchkiss Brain Institute, University of Calgary, 2500 University Drive NW, Calgary, AB, Canada T2N 1N4

## Abstract

The long-term effects of cannabis on human cognition are still unclear, but, considering that cannabis is a widely used substance and, overall, its potential use in therapeutic interventions, it is important to evaluate them. We hypothesize that the discrepancies among studies could be attributed to the specific cognitive function investigated and that skills subserved by the hippocampus, such as the spatial orientation abilities and, specifically, the ability to form and use cognitive maps, should be more compromised than others. Indeed it has been showed that cannabis users have a reduced hippocampus and that the hippocampus is the brain region in which cannabis has the greatest effect since it contains the highest concentration of cannabinoid receptors. To test this hypothesis we asked 15 heavy cannabis users and 19 nonusers to perform a virtual navigational test, the CMT, that assesses the ability to form and use cognitive maps. We found that using cannabis has no effect on these hippocampus-dependent orientation skills. We discuss the implications of our findings and how they relate to evidence reported in the literature that the intervention of functional reorganization mechanisms in cannabis user allows them to cope with the cognitive demands of navigational tasks.

## 1. Introduction

Cannabis is a substance widely abused in the world [1, 2], but it can be included in a variety of therapeutic interventions (e.g., in the management of multiple sclerosis) [3]. Therefore, it is crucial to evaluate the effects of this substance on cognitive and neural functioning. Studies which evaluated the acute effects of cannabinoids showed that cannabis affects different cognitive skills, including short- and long-term memory, learning, and executive functions [4–7]. By contrast, the findings of studies that investigated the long-term effects of cannabis use were inconsistent and, thus, failed to provide a clear understanding of the effects of long-term cannabis use on human cognition. Specifically, some studies reported differences in memory, learning, executive functions, and attention when cannabis users were compared with nonusers [8–15]; and other studies reported no persistent cannabis-related cognitive deficits on different kinds of tasks [16–21]. Moreover, functional magnetic resonance imaging studies in cannabis users have usually reported increased BOLD signals in additional brain regions as compared with nonusers during performance of a variety of cognitive tasks. Despite this additional cluster of neural mechanisms in cannabis users, differences in behavioral performance between user and nonusers have been hardly ever detected (for a review see [22]). The latter observation suggests that the brain may be capable of functional reorganization through the activation of brain regions that are not adopted in nonusers to cope with the cognitive demand [22].

Various factors, including the length of abstinence periods prior to testing [19], could be responsible for the differences among studies investigating the long-term effects of cannabis use. Consistent with this observation, we found that some studies evaluated users' cognitive performance after a long abstinence period (i.e., one month [11, 13, 23]), whereas other studies assessed cognitive performance after 12–15 hours of abstinence [9, 15]. Pope and colleagues [23] showed that heavy cannabis users scored worse than nonusers on tasks requiring the recall of word lists; this effect was present for seven days after cannabis use and disappeared 28 days after cannabis use, suggesting that length of abstinence before testing may be a critical factor in detecting cognitive impairments in this population. Nevertheless, this factor cannot account for studies (in which cannabis users were tested a few hours after cannabis use) that failed to find a significant difference in the performance of users and nonusers (e.g., [19]).

Other factors that might explain contrasting results on the long-term effects of cannabis might be related to specific parameters of use such as onset, frequency, and duration. However, there are also studies that failed to find a correlation between these parameters and the cognitive performance of cannabis users (e.g., [15]).

Perhaps the incongruity among studies can be better explained by the specific cognitive functions assessed. For example, there is evidence that attention and memory functions are more compromised than other cognitive skills [24, 25]. Also within the specific memory domain, it has been suggested that cannabis use affects some components (i.e., episodic memory) more than others (i.e., implicit memory and working memory) [4, 19].

Therefore, if (as it seems) the cognitive function evaluated accounts for the inconsistency among studies, it would be interesting to investigate cognitive skills that have not yet been studied in cannabis users and that might be compromised. Some studies found the highest density of cannabinoid receptors (CB1) in the hippocampus [26, 27]; furthermore, a bilateral volumetric reduction of the hippocampus has been reported in both adult [28, 29] and adolescent [30] heavy cannabis users. Thus, cognitive functions in which the hippocampus plays an important role might be more impaired than others.

 Spatial memory impairments, such as getting lost or forgetting where objects are located, are a common consequences of hippocampal damage in humans [31]; moreover, the cells that encode spatial information (i.e., “place cells”) have been found in the hippocampus of both humans [32] and animals [33]. Furthermore, among the theories regarding the role of the hippocampus in memory, the Cognitive-Map Theory [34] is widely accepted among scientists [31]. In this theory, the hippocampus is considered a critical structure for visuospatial and environmental memory because it constructs a representation of the environment, that is, a cognitive map, in which different landmarks (and their spatial relationships) are represented. Using cognitive maps for orientation is critical because they allow reaching any target location starting from any place and by following any route available within the environment. The basic role of the hippocampus in establishing and maintaining a cognitive map is supported by the findings of several neuropsychological [32, 35–39] and fMRI studies [40–43].

As cannabis users show volumetric reduction of the hippocampus [28, 29], it is reasonable to speculate that they may have a cognitive deficit in hippocampal-dependent cognitive skills. Therefore, assessing the ability of cannabis users to orient by means of cognitive maps could contribute to clarifying the long-term effects of using this substance.

In the present study, we asked cannabis users and nonusers to perform a task previously developed to investigate the ability to form and use cognitive maps: the Cognitive Map Test (CMT) [42]. Performance on the CMT depends on hippocampal functioning [42] and hippocampal structural integrity [44] in healthy controls (non-cannabis users). Specifically, neuronal activation was found in the anterior hippocampus during the formation of a cognitive map and in the posterior hippocampus during the use of a cognitive map. At a deeper level of analysis, as cannabis users show greater volumetric reduction of the right anterior hippocampus [28], we hypothesized that they would have a pervasive deficit in the ability to form a cognitive map.

## 2. Methods

### 2.1. Participants

We recruited 43 men: 24 were cannabis users (Users) and 19 were drug-naive healthy subjects (Naives). As only a few women responded to our research advertisement and due to the well-known differences in navigational abilities between the sexes [45–49], we decided to include only men in the study. The participants did not report major medical, neurological, or neuropsychiatric diseases or use of psychotropic medication. All participants gave their written informed consent to take part in the study, which was approved by the local Ethics Committee.

The following criteria were used to select the user participants: the only abusive substance they used was cannabis; they had used cannabis regularly (5–7 days/week) for the last two years; they had abstained from using cannabis for no less than 12 hours prior to testing.

We excluded five individuals from the sample who reported using other illicit recreational drugs (MDMA and cocaine), two who had given up smoking cannabis one year prior to being tested, and two who used cannabis occasionally (lifetime use of less than 250 joints). The final sample consisted of 15 heavy cannabis users (lifetime exposure range: 965–23040 joints) and 19 non-cannabis users.

### 2.2. Experimental Protocol

Participants were asked to fill in a questionnaire aimed at collecting information about the use of cannabis and other recreational drugs, as well as legal substances.

Unfortunately, due to limited resources we were unable to provide an objective measure of recent drug use (i.e., from hair or urine samples). According to Fisk and Montgomery [50], this is clearly a limitation; nevertheless, it is not without precedents [50–54], and we have no reason to doubt the truth of the information provided by the participants because they received no reward for taking part in the study. In any case, this limitation must be considered when we discuss the results.

 After filling in the questionnaire, the participants were asked to perform a reading test in order to measure their IQ (TIB-Test d'intelligenza Breve, *Brief intelligence test*) [55] and to complete the Beck Depression Inventory (BDI) [56] to determine whether they had mood disorders.

Finally, the participants were asked to perform the Cognitive Map Test (CMT), which assesses the ability to form and use cognitive maps in virtual environments [42]. The experimental paradigm consisted of a virtual city composed of several buildings of different sizes and shapes but of the same texture. The city included six landmarks: a cinema, a restaurant, a bar, a hotel, a pharmacy, and a flower shop. Participants navigated in the city by using a three-button keypad; each button corresponded to movement in one of three directions: left, forward, and right.

Before testing, participants were submitted to a practice phase in which they navigated freely for five minutes in a city similar to the experimental one. Then, they were asked to perform control trials in which they had to navigate, as quickly as possible, a route indicated by arrows. These control trials were performed to ensure that all participants were comfortably moving around the environment using the directional buttons. The experimental tasks started when the participants finished the three consecutive control trials without stopping along the designated pathways.

**Figure 1:**
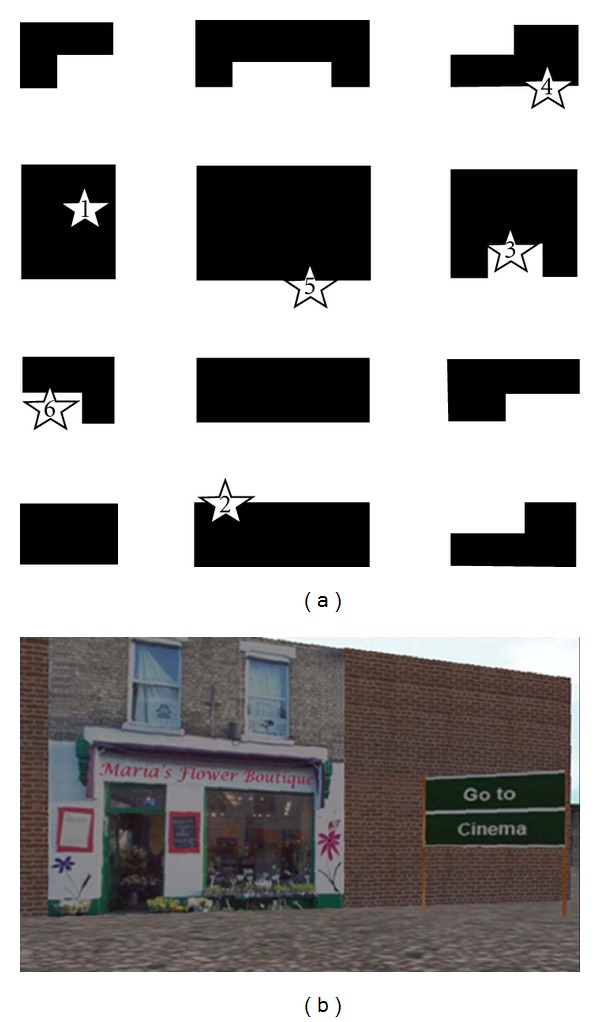
Cognitive Map Test. (a) Schematic map of the city. Numbers indicate the location of the landmarks (1: cinema; 2: flower shop; 3: hotel; 4: bar; 5: pharmacy; 6: restaurant). (b) View of the participants' starting position in one trial of the retrieval task.

The experimental test consisted of two tasks: learning and retrieval. During the learning task, participants were instructed to freely explore the environment in order to create a mental representation of the city, including the location of the six landmarks. After six minutes, the examiner asked the participants to report the locations of the six landmarks on a schematic map representing the city from a top-view perspective (Figure 1(a)). If they made errors in locating the landmarks, they had to explore the virtual city for another minute, at the end of which they were again requested to locate the landmarks on a new map. The participants were allowed to perform 24 one-minute sessions, after which the investigator stopped the task. The learning task was considered completed when the participants were able to indicate the correct locations of all six landmarks, or at the end of trial 25 (i.e., after 30 minutes of learning). All participants completed the task in 25 trials. We measured the time spent to perform the task, that is, the time required to form the cognitive map of the environment.

The retrieval task was administered soon after completion of the learning task and consisted of 18 trials in which participants were asked to reach specific landmarks by relying on the cognitive map they had just formed. In each trial, the starting position faced one of the six landmarks, and a written signal indicated the target location the participants had to reach as quickly as possible following the shortest route (Figure 1(b)). The duration of each trial was recorded as a measure of behavioral performance.

## 3. Results

Users and Naives were matched for age (*t*_1,32_ = 0.97, n.s.), years of education (*t*_1,32_ = 1.84, n.s.), and estimated premorbid intelligence (*t*_1,32_ = 1.8, n.s.). Both groups of participants made similar weekly use of tobacco (*t*_1,32_ = −1.68, n.s.) and alcohol (*t*_1,32_ = −1.40, n.s.). Users scored higher on a depression scale (*t*_1,32_ = −2.4, *P* = 0.02), consistently with the findings of previous studies that cannabis users have more depressive symptoms than nonusers [29, 57, 58].

See Table 1 for participants' demographic and cannabis use information.

Separate *t*-test analysis revealed no significant differences between groups in either CMT-learning (Users: mean = 556 sec., SD = 149.51; Naives: mean = 546.31 sec., SD = 226.18; *t*_1,32_ = −0.14; n.s.) or CMT-retrieval (Users: mean = 355.4 sec., SD = 105.7; Naives: mean = 315.11 sec., SD = 99.98; *t*_1,32_ = −1.14; n.s.) task. Moreover, a qualitative analysis of performance showed that all participants were able to reach the target locations in the CMT-retrieval task by following the shortest path. These data confirm that both groups of participants were able to form a cognitive map of the environment and to use it for navigational purposes.

No significant correlation between the CMT (learning and retrieval tasks) and lifetime cannabis exposure, that is, number of joints (*r* = −0.001, learning task; *r* = 0.19, retrieval task) or length of use (*r* = −0.018, learning task; *r* = 0.24 retrieval task), was observed. This suggests that these specific parameters of use had no effect on the ability to orient using cognitive maps.

## 4. Discussion

Recent studies show bilateral volumetric reduction of the hippocampus in chronic cannabis users [28, 29]. This finding is consistent with the high concentration of cannabinoid receptors (CB1) found in the hippocampus [26, 27] and reports that, after long-term exposure to THC, the number of neurons and synapses in the hippocampus were decreased [59, 60]. These hippocampal changes may constitute a morphological basis for behavioral effects of long-term cannabis exposure. Hence, cognitive functions in which the hippocampus plays a critical role, such as spatial orientation and navigation [35–37, 39–43], could be particularly compromised.

This is the first study that has investigated the ability of heavy cannabis users to form and use cognitive maps, that is, cognitive skills that have been shown to involve the hippocampus [35–37, 42]. Our data showed no significant difference between cannabis users and nonusers on these navigational tasks.

Many studies, however, have described a strict relationship between cannabis use and low cognitive performance [8, 9, 24, 61, 62]. One explanation of this discrepancy could lie in the different parameters of cannabis use (i.e., lifetime exposure and length of use) between participants in our study and in previous studies. Indeed, Messinis and colleagues [24] examined the neuropsychological functioning of 20 long-term and 20 short-term heavy frequent cannabis users and found that specific cognitive domains declined with increasing years of heavy, frequent cannabis use. Nestor and coworkers [15], however, found no differences in performance related to specific parameters of cannabis use. Interestingly, Battisti and colleagues [9] showed worse performance on a verbal memory test in short-term regular users of cannabis compared with long-term users and hypothesized that prolonged use might lead to neuroadaptation to compensate for cognitive dysfunctions. In sum, the differences among studies could be due to differences in the parameters used. Paradoxically, in some cases heavy cannabis users with a history of long-term use performed better than short-term users thanks to the intervention of neuroadaptation processes. In our study, we included subjects who were not occasional users and who had different lengths of use. If cumulative cannabis exposure and length of use are responsible for worse performance, we should have observed reduced performance in participants with longer use, which was not the case. On the other hand, if the absence of cognitive impairments is due to neuroadaptation, we should have found worse performance in participants with shorter use, which, again, was not the case. Instead, our findings revealed that the ability to form and use cognitive maps is not related to parameters of use such as lifetime exposure to cannabis and length of use. Moreover, the fact that the cannabis users who participated in this study scored higher on the depression scale is consistent with the findings of previous studies [29, 57, 58] and could indicate that our sample exhibited characteristics typical of a heavy-user population.

The lack of group differences in creating and using a cognitive map could have been due to the low difficulty of our experimental tasks. Indeed, cannabis users would probably be more impaired on cognitively challenging tasks. However, we think that this was not the case. Indeed the CMT is not an easy task, and previous studies have showed that it is sensitive in detecting gender differences in healthy individuals (healthy women need twice as much time as men to learn a cognitive map) [48], navigational difficulties in individuals with developmental topographical disorientation [63, 64], and age differences between young and older healthy individuals [65].

In any case, our results are in line with those of studies in which the performance of cannabis users did not differ from that of nonusers on other cognitive tasks relying on the hippocampus [19, 30]. For instance, Jager and colleagues [19] reported no differences between cannabis users and nonusers in the ability to perform a hippocampal-dependent associative memory task, even though reduced BOLD signal changes were detected in the users' hippocampal complex during the fMRI study.

Moreover, also some studies on the acute effects of cannabis on specific cognitive abilities showed that, while the behavioral performance was not significantly modulated by THC administration, the activation of specific brain areas was augmented or attenuated compared to the condition in which a placebo was administrated [66–68].

 This dissociation between neuroimaging and behavioral data suggests that cannabis users may rely on different neural mechanisms to perform a cognitive task. In other words, as suggested by Martín-Santos and colleagues [22], in the cannabis-user population the brain might be capable of functional reorganization through recruitment of additional regions to cope with the cognitive demand.

Unlike previous studies, ours is the first one that investigated navigational skills, which are involved in most daily life activities. Using cannabis is considered to have a progressive effect on cognitive domains and the hippocampus [24, 29, 61, 62], and it is possible that daily life exercise of navigational skills facilitates the plastic reorganization of these functions and that users come to rely heavily on extrahippocampal brain regions. Consistent with this speculation is the fact that our experimental sample included only men. Several studies on spatial orientation have shown that forming and using cognitive maps is the preferred navigational strategy in men [48, 69]. This suggests that men are more likely to exercise the abilities tested by the CMT in daily life. This reorganization, however, should not be activated, or should be less effective, for cognitive tasks used less frequently in daily life and should account for the reported low performance on tests that, for example, require memorizing lists of words or connecting circles in an ascending pattern.

## Figures and Tables

**Table 1:** Participants' demographic information and drug use.

Measure	Users	Naives	*t*-test
Age (years), mean (SD)	25.07 (3.43)	26.21 (3.37)	*t*_1,32_ = 0.97 (n.s.)
Education (years), mean (SD)	13.47 (2.03)	14.84 (2.26)	*t*_1,32_ = 1.84 (n.s.)
IQ, mean (SD)	112.98 (3.45)	114.89 (2.78)	*t*_1,32_ = 1.8 (n.s.)
BDI*, mean (SD)	6.53 (5.72)	3 (2.62)	*t*_1,32_ = −2.4 (*P* = 0.02)

Cannabis use			

Lifetime exposure (no. of joints), mean (SD)	6119 (6346.83), (range: 965–23040)	—	
Use during previous 7 days (joints), mean (SD)	17.93 (18.44)	—	
Use during previous 30 days (joints), mean (SD)	73.47 (57.89)	—	
Cannabis length of use (years), mean (SD)	7.13 (3.33)	—	
Average weekly dose (joints), mean (SD)	16.75 (11.54)	—	

Tobacco smoking (no. of cigarettes/week), mean (SD)	75.26 (82.07)	41.41 (50.33)	*t*_1,32_ = −1.68 (n.s.)
Alcohol units** (no. of units/week), mean (SD)	8.13 (8.65)	4.89 (4.59)	*t*_1,32_ = −1.4 (n.s.)

*Beck Depression Inventory (BDI).

**A unit was constituted by a glass of wine, a single measure of spirit or a small beer (250 mL).
